# Analysis on Acupoint Selection and Combination for Amyotrophic Lateral Sclerosis Treated with Acupuncture Based on Data Mining

**DOI:** 10.1155/2022/6541600

**Published:** 2022-06-08

**Authors:** Jia Xu, Zhengyu Lu, Hongjing Zhang, Yan Shen, Hong Zhao

**Affiliations:** ^1^VIP Department, Longhua Hospital, Shanghai University of Traditional Chinese Medicine, Shanghai 200032, China; ^2^Teaching Affairs Department, Yueyang Hospital of Integrated Traditional Chinese and Western Medicine, Shanghai University of Traditional Chinese Medicine, Shanghai 200437, China

## Abstract

**Objective:**

The aim of the study was to explore the regularity of acupoints in the treatment of amyotrophic lateral sclerosis (ALS) by means of data mining technology.

**Methods:**

Nine databases, including SinoMed, Chongqing VIP (CQVIP), China National Knowledge Infrastructure (CNKI), Wanfang Data, Cochrane Library, PubMed, MEDLINE, Web of Science, and Embase, were comprehensively searched till December 2021. The published clinical literature testing acupuncture in the treatment of ALS was eligible for inclusion. Studies were organized to establish the prescription database. Modular data mining analysis, including acupoint frequency, complex network analysis, association rule analysis, and cluster analysis were used to conduct statistical analysis.

**Results:**

Forty-two literature studies on 141 acupoints were included, involving 626 times the total application frequency. The top 5 acupoints in application frequency were Hegu (LI 4, 67%), Zusanli (ST 36, 67%), Quchi (LI 11, 52%), Sanyinjiao (SP 6, 48%), and Yanglingquan (GB 34, 45%). The most involved meridian was the large intestine meridian of hand Yangming (90 times). The generally used acupoints were mainly distributed in the lower limbs. The top 5 combinations in application frequency were Hegu-Quchi (75 times), Quchi-Zusanli (66 times), Zusanli-Sanyinjiao (54 times), Hegu-Sanyinjiao (54 times), and Quchi-Sanyinjiao (49 times). The acupoint combinations with the strongest association were Quchi, Hegu, Zusanli, Sanyinjiao, and Shousanli (LI 10). There were 7 acupoint groups according to the cluster analysis. The core prescriptions were Hegu, Zusanli, Quchi, and Jiaji (EX-B 2).

**Conclusions:**

Hegu, Zusanli, Quchi, and Jiaji could be used as the main prescriptions in treating ALS. The combination of Quchi, Hegu, Zusanli, and Sanyinjiao should be selected with priority in acupuncture therapy.

## 1. Introduction

Amyotrophic lateral sclerosis (ALS) is an adult-onset devastating disease with substantial socioeconomic burden. As the most common motor neuron disease, ALS is perceived as a complex polygenetic neurodegenerative disorder involving the upper and lower motor neuron compartments, which is characterized by progressive degeneration of motor neurons in the cortex, corticospinal tract, brainstem tract, and anterior horn of the spinal cord [1, 2]. Affected areas include the anterior horn cells in the spinal cord and the motor nuclei in the medulla oblongata and pons. Patients experience signs and symptoms of progressive muscle atrophy, which occurs on the thenar muscles initially [3], and eventually die of respiratory failure [4], with a median survival of 2–4 years from the onset [5]. As of 2010, ALS had a prevalence of 1.0 per 100,000 persons in China, with 6,170 incident cases every year [6]. Notably, the prevalence is estimated to increase by almost a half from 2015 to 2040 in China [7]. Riluzole is the first drug as a glutamate release inhibitor approved by the United States Food and Drug Administration (FDA), for expansion of life by 3–5 months [8]. Another FDA-approved medical treatment is edaravone, which is simultaneously recommended as an antioxidant for the use of recovery from stroke. There currently remains no evidence-based disease-modifying therapy for this terrible condition. Patients and their families seek for some complementary and alternative therapies urgently to clinically improve the quality of life. Through the ages, people have realized the curative benefits of natural products. Several ethnomedicinal herbs have been promoted to equip a neurobehavioral state and operate as a complement to modern medications [9, 10]. In addition to improving physical conditions [11], these natural bioactive compounds play an active modulatory role in the pathological molecular mechanisms of neurodegenerative disorder development [12, 13], such as ALS, with the potential neuroprotective pharmaceutical value [14].

Acupuncture, since ancient times, as an adjunctive therapy, has been commonly used in patients with ALS in China. In Shanghai, approximately 51.32% of the patients used acupuncture therapy to treat weakness and muscle atrophy, to delay the development of ALS, and to deal with depression, insomnia, poor appetite, and side effects of riluzole [15]. Acupuncture is a unique nonpharmacological treatment that protects neurons from degeneration and promotes axonal regeneration in neurodegenerative diseases such as ALS [16]. Acupuncture activates endogenous opioid peptides with evidence of the modulation of the immune system [17, 18], which could alter the progression of diseases theoretically, where the immune system plays a pathogenic role, including ALS [19]. Some researchers find varied inflammatory markers in ALS animal models through electroacupuncture [20]. In doing so, earlier acupuncture may improve the life quality and prolong the survival duration [1]. Currently, there is still no systematic review focusing on acupuncture therapy in treating ALS clinically. In traditional Chinese medicine (TCM), the acupoint selection and combination play a vital role in the significant effect of acupuncture therapy. However, determining the optimal acupoints for ALS still remains to be elucidated. With the rapid development of big data, data mining techniques that use algorithms, including classification, association, and clustering rules, are significant statistical exploratory data analysis tools that have been widely applied to investigate hidden concepts in relation within big datasets [21].

In this study, we aimed to explore the regularity of acupoint selection and combination based on the acupuncture literature in treating ALS by means of data mining techniques with the purpose of informing the selection of optimal acupoints in clinical practice for the treatment of ALS.

## 2. Materials and Methods

### 2.1. Data Sources and Search Strategy

A comprehensive search was performed up to December 2021 with no language restriction, including 9 databases (Cochrane Library, PubMed, Embase, Web of Science, MEDLINE, SinoMed, CQVIP, CNKI, and Wanfang Data), using medical subject heading terms (MeSH) in the whole process. Besides, the reference lists of all relevant studies were verified for related citations to ensure the integrality of the search.

The following MeSH terms were used in the conduct of a search, including (“amyotrophic lateral sclerosis” OR “motor neuron disease” OR “ALS” OR “MND”) AND (“Acupuncture Therapy” OR “acupuncture” OR “electroacupuncture” OR “manual acupuncture” OR “acupoint” OR “meridian” OR “scalp acupuncture” OR “elongated acupuncture” OR “abdominal acupuncture”). Search strategies are presented in Supplementary Appendix 1.

### 2.2. Eligibility Criteria

Clinical literature studies were included in this review according to the following criteria: (1) were performed in ALS patients diagnosed with the EI Escorial criteria of the World Federation of Neurology by Electromyogram [22, 23], without any restriction on age or gender; (2) evaluated the effects of acupuncture (manual acupuncture, electroacupuncture, etc.) compared with a control group (sham acupuncture or medical therapy); and (3) included at least one of the following outcomes: ALS functional rating scale-revised scores, the modified Norris Scale, ALS Assessment Questionnaire-40, and the effective rate.

In terms of the exclusion criteria, the following studies were excluded: (1) repeated publications (only extracting data from the recent publication); (2) reviews, systematic reviews, meta-analysis, or animal experiments; and (3) trials only stimulating nonacupoint trigger points based on physiology and anatomy.

### 2.3. Data Extraction

We screened the studies according to the criteria. Duplicate and irrelevant articles were eliminated through titles and abstracts. After reading the full text, we extracted data from the eligible literature in a standardized data form using the SPSS 25.0 software (SPSS Inc., Chicago, IL, USA). Data including author information, publication year, TCM syndrome types, intervention, comparator, acupoints, and outcomes were extracted. The description of meridians and acupoints in the included data was standardized and unified according to WHO Standard Acupuncture Point Locations in the Western Pacific Region [24]. Two reviewers performed the work independently. Missing or conflicting data were discussed and managed by the third author.

### 2.4. Data Processing

Information about acupoints and combination was conducted via the ancient and modern medical case cloud platform (V2.3.5), which was developed by China Academy of Chinese Medical Sciences on the evidence of nearly 400,000 TCM literature. We conducted modular data mining analysis, including acupoint frequency, cluster analysis, association rule analysis, and complex network analysis to obtain the optimal acupoints and combination.

## 3. Results

### 3.1. Summary of Included Studies

**Figure 1:**
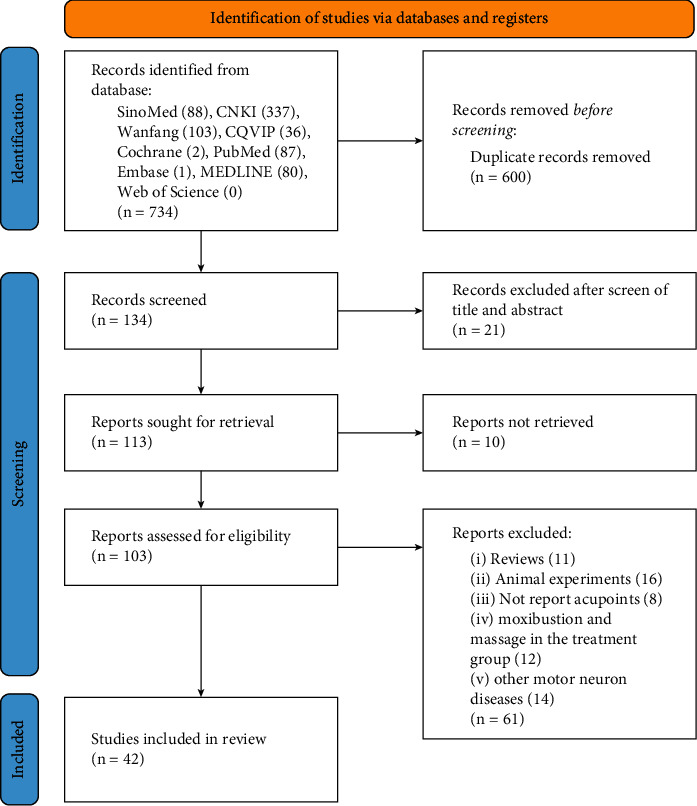
Flowchart of the study selection process.

There were a total of 734 relevant articles through the search strategy previously described. Database searching identified 564 records in CNKI, SinoMed, Wanfang, and CQVIP and 170 records in PubMed, Cochrane, MEDLINE, and Embase. After eliminating duplicates, 134 papers were included for further screening, of which 42 eligible studies were included according to the criteria and thus subjected to our analysis (Figure 1). All included papers were published from 1999 to 2021. One study [25] was conducted in Korea, another one study [26] was conducted in Australia, and the others were conducted in China.

### 3.2. Descriptive Analysis

**Figure 2:**
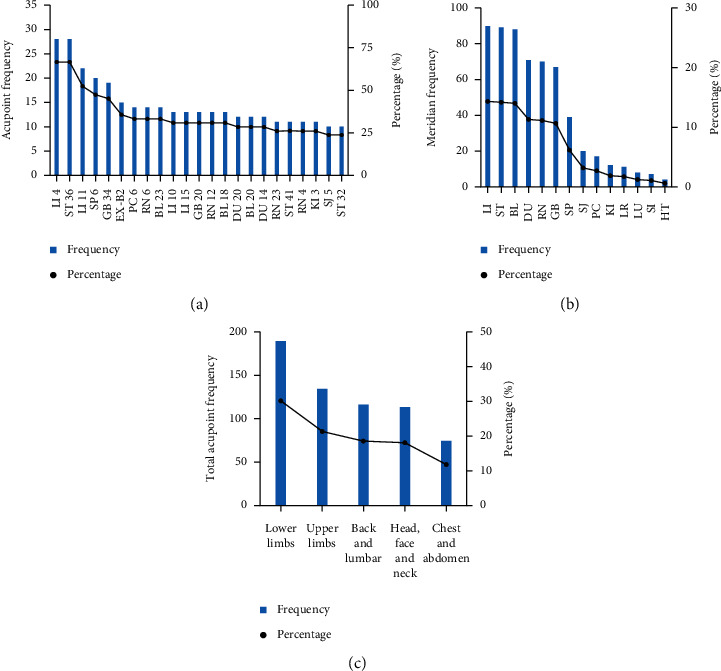
The frequencies of the acupoints and meridians. (a) Acupoints (>10 times) in the treatment of ALS; (b) meridian distribution associated with ALS; (c) body part distribution of acupoints.

Acupoint frequency was analyzed based on the eligible 42 acupoint prescriptions. There were 141 acupoints involved in the treatment of ALS, which were recorded in total 626 times. The top 5 acupoints used frequently were found to be Hegu (LI 4, 67%), Zusanli (ST 36, 67%), Quchi (LI 11, 52%), Sanyinjiao (SP 6, 48%), and Yanglingquan (GB 34, 45%) in turn (Figure 2(a)).

There were 131 meridian acupoints and 10 extra points (33 times) in the 42 acupoint prescriptions. The most involved meridian was found to be the Large Intestine Meridian of Hand Yangming (LI, 90 times), followed by the Stomach Meridian of Foot Yangming (ST, 89 times) and the Bladder Meridian of Foot Taiyang (BL, 88 times), which came in at 43% together for total frequencies up to 267 records. Fifty-four acupoints were ascribed to the 3 meridians, which accounted for 38% of the total acupoint number. Futhermore, Yang meridians (58%) were used more frequently compared to the Yin meridians (15%). Notably, Jiaji (EX-B2), as an extra point, was recorded 15 times, which accounted for 46% of the total extra point number. The distribution of meridians is shown in Table 1 and Figure 2(b).

Acupoints on the lower limbs were used most frequently with a total frequency of 189 times, followed by acupoints on the upper limbs (134 times), the back and lumbar (116 times), the head, face, and neck (113 times), and the chest and abdomen (74 times). The most involved acupoints were found on the head, face, and neck, which accounted for 23% of the total acupoint number (Table 2, Figure 2(c)).

### 3.3. Complex Network Analysis

A total of 44 acupoint combinations were obtained from all acupoints by means of complex network analysis, in which the minimum edge weight was set to 30. The top 10 most frequently used combinations were [LI4]–[LI11], [LI11]–[ST36], [ST36]–[SP6], [LI4]–[SP6], [LI11]–[SP6], [RN6]–[LI4], [LI4]–[GB34], [ST36]–[GB34], [RN6]–[ST36], and [LI4]–[LI15] (Supplementary Appendix 2).

### 3.4. Association Rule Analysis

**Figure 3:**
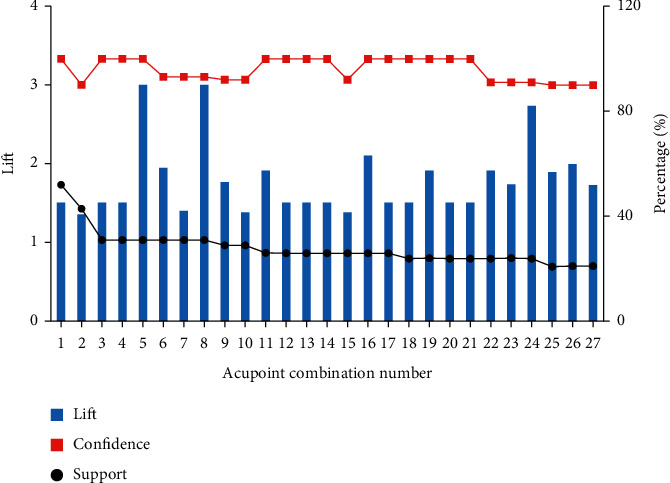
Association rule analysis of acupoint compatibility.

We obtained a total of 27 acupoint association rules from 23 acupoints (>10 times) for ALS using the apriori algorithm, in which the minimum support required was set to 15% and the minimum confidence required was set to 90% (Figure 3). The lift should be bigger than 1 for a rule to be a positive association. Among these rules, the lift ranged from 1.35 to 3.0. On these grounds, the top 10 most frequent acupoint combinations were [LI11]–[LI4, ST36], [SP6]–[LI4, ST36], [LI10]–[LI4, ST36], [LI15, LI10]–[LI4], [BL18]–[BL23], [RN6]–[SP6], [BL23]–[ST36], [BL23]–[BL18], [LI10, LI15]–[LI11], and [LI15, BL18]–[ST36] (Supplementary Appendix 3).

### 3.5. Hierarchical Cluster Analysis

**Figure 4:**
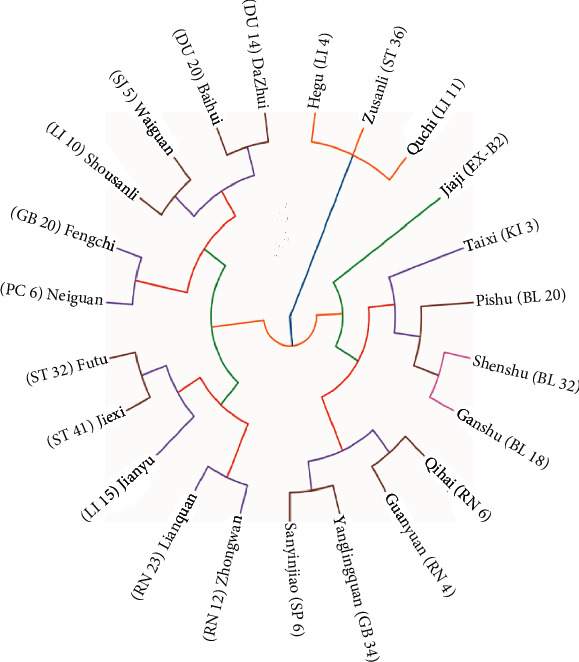
Hierarchical clustering dendrogram of acupoints for ALS.

Through Euclidean distance and Ward's maximum variance method, acupoints with a frequency greater than 10 were clustered into 7 major clusters: Cluster 1 included LI4, ST36, and LI11; Cluster 2 included EX-B2; Cluster 3 included KI3, BL20, BL23, and BL18; Cluster 4 included RN6, RN4, GB34, and SP6; Cluster 5 included RN12, RN 23, LI15, ST 41, and ST32; Cluster 6 included PC6 and GB20; and Cluster 7 included LI10, SJ5, DU20, and DU14 (Figure 4).

## 4. Discussion

In this study, data mining analysis was used to identify acupoint selection and combination based on 42 acupoint prescriptions in the clinic. It provided preliminary evidence for acupuncture therapy to treat ALS.

In TCM, the earliest medical classic, *Huangdi's Internal Classic of Medicine*, has a record about this syndrome, in which the meridians of Yangming are widely used for its treatment. The results showed that LI (14.38%), ST, and BL were the most frequently used meridians in these eligible clinical studies, which conformed to the principles of the ancient classics. According to the meridian circulation, LI starts from the radial tip of the index finger, running upward along the finger, the anterior aspect of the forearm, the elbow, the upper arm, and the shoulder in turn, which is related to its indications on the weakness and muscle atrophy of the upper limbs in treating ALS. BL starts from the head, related to the brain, and dominates the main skeletal muscles involved in the movement of the lower limbs, which could be applied in treating motor system diseases, such as ALS. Moreover, acupoints on the lower limbs (30%), particularly in Yang meridians, were used most frequently, such as Zusanli (ST36), Sanyinjiao (SP6), Yanglingquan (GB34), and Jiexi (ST41), which are distributed around the joints.

According to the frequency, the top 5 acupoints were found to be Hegu (LI 4), Zusanli (ST 36), Quchi (LI 11), Sanyinjiao (SP 6), and Yanglingquan (GB 34) in turn. The top 5 most frequent acupoint combinations were [LI4]–[LI11], [LI11]–[ST36], [ST36]–[SP6], [LI4]–[SP6], and [LI11]–[SP6]. The interconnections between the acupoints indicated that LI 4, LI 11, ST 36, and SP 6 were the key node acupoints. The results of association rule analysis and complex network analysis were consistent with each other. The combinations of [LI11]–[LI4, ST36], [SP6]–[LI4, ST36], and [LI10]–[LI4, ST36] appeared to be of high support. Previous evidence illuminated that acupuncture on ST 36, LI11, and LI4 exerts a beneficial effect on the promotion of neurogenesis and cell proliferation in the central nervous system [27]. Besides, earlier electroacupuncture at ST 36 in ALS mice is related to the effects on the suppression of nerve inflammation by reducing the activation of small and medium-sized glial cells in the brain stem and the spinal cord [28]. Electroacupuncture at LI 11 and ST 36 plays a neuroprotective role in the improvement of the motor function by promoting the secretion of M2 of microglia exosomes [29].

In the clinical practice of acupuncture therapy, acupoint selection and combination according to the theory of meridians and collaterals as well as syndrome differentiation in TCM are of vital importance to the therapeutic effects [21]. Using hierarchical cluster analysis, 7 major clusters were obtained from the eligible acupoints. The first 2 clusters, including Hegu (LI 4), Zusanli (ST 36), Quchi (LI 11), and Jiaji (EX-B2), were the main acupoints in treating ALS. EX-B2 are extra nerve acupoints adjacent to governor vessels and dorsal shu points, which are related to the spinal cord. When conducting electroacupuncture at EX-B2, the highly upregulated Sema3A and neuropilin 1 were reversed postspinal cord injury, which can reduce the accumulation of peripheral nerve networks around the central tube of the spinal cord gray matter and promote the recovery of motor functions in rats [30]. Furthermore, the other 5 clusters were defined as the preliminary adjunct acupoints. In particular, Taixi (KI 3), Shenshu (BL 23), Ganshu (BL 18), and Pishu (BL 20) could be used to treat deficiency of liver and kidney syndrome manifested by the weakness of the lower limbs, dizziness, and tinnitus. Qihai (RN 6), Guanyuan (RN 4), and Sanyinjiao (SP 6) could be applied to treat deficiency of spleen and stomach syndrome manifested by fatigue and poor appetite. Dazhui (DU 14), Waiguan (SJ 5), and Baihui (DU 20) could be added to treat fever, sweating, thirst, and upset. Lianquan (RN 23) and Zhongwan (RN 12) could be used to treat central bulbar paralysis, while Neiguan (PC 6) and Fengchi (GB 20) are applied in dementia patients.

Some limitations in the study should be considered. First, the current publication number of acupuncture therapy on ALS affected the sample size of this data mining analysis. Although the rapid progress of ALS limits the implementation of clinical research, further large clinical evidence of high quality is urgently needed. Second, since the study focuses on the main acupoints in the treatment of ALS, data regarding adjunct acupoints were not extracted for analysis. In the future, these data could be further mined to identify more synthetical acupoint selection patterns based on TCM syndrome differentiation theory.

## 5. Conclusion

In conclusion, acupoints on the Large Intestine Meridian of Hand Yangming, distributed in the lower limbs are the main points for ALS treated with acupuncture. Hegu (LI 4), Zusanli (ST 36), Quchi (LI 11), and Jiaji (EX-B2) are the main prescriptions in syndrome differentiation. The combinations of Quchi (LI 11), Hegu (LI 4), Zusanli (ST 36), and Sanyinjiao (SP 6) are defined as the potential combinations that should be selected with priority in acupuncture therapy. Overall, our paper provides a preliminary suggestion for the optimal acupoint selection and combination in the acupuncture treatment of ALS clinically.

## Figures and Tables

**Table 1:** Frequency and percentage of acupoints used for ALS.

Meridians	Frequency	Percentage	Acupoint number	Acupoints (frequency)
LI	90	0.14	12	Hegu (LI 4) (28), Quchi (LI 11) (22), Shousanli (LI 10) (13), Jianyu (LI 15) (13), Yangxi (LI 5) (4), Sanjian (LI 3) (2), Shouwuli (LI 13) (2), Binao (LI 14) (2), Pianli (LI 6) (1), Wenliu (LI 7) (1), Futu (LI 18) (1), Erjian (LI 2) (1)
ST	89	0.14	20	Zusanli (ST 36) (28), Jiexi (ST 41) (11), Futu (ST 32) (10), Biguan (ST 31) (8), Liangqiu (ST 34) (8), Fenglong (ST 40) (4), Tianshu (ST 25) (4), Qichong (ST 30) (3), Tiaokou (ST 38) (2), Xiangu (ST 43) (2), Jiache (ST 6) (1), Wailing (ST 26) (1), Touwei (ST 8) (2), Huaroumen (ST 24) (2), Xiaguan (ST 7) (2), Neiting (ST 44) (2), Quepen (ST 12) (1), Chongyang (ST 42) (1), Shangjuxu (ST 37) (1), Dubi (ST 35) (1)
BL	88	0.14	22	Shenshu (BL 23) (14), Ganshu (BL 18) (13), Pishu (BL 20) (12), Weishu (BL 21) (6), Weizhong (BL 40) (6), Kunlun (BL 60) (5), Feishu (BL 13) (4), Xinshu (BL 15) (4), Tianshu (BL 10) (4), Geshu (BL 17) (3), Chengshan (BL 57) (2), Luoque (BL 8) (1), Tongtian (BL 7) (1), Pohu (BL 42) (1), Shentang (BL 44) (1), Guanyuanshu (BL 26) (1), Chengguang (BL 6) (1), Ciliao (BL 32) (1), Wuchu (BL 5) (1), Geguan (BL 46) (1), Zhibian (BL 54) (1)
DU	71	0.11	18	Baihui (DU 20) (12), Dazhui (DU 14) (12), Mingmen (DU 4) (6), Shuigou (DU 26) (6), Shenting (DU 24) (5), Fengfu (DU 16) (4), Yaoyangguan (DU 3) (4), Yamen (DU 15) (3), Shenshu (DU 12) (3), Zhongshu (DU 7) (2), Jinsuo (DU 8) (2), Yaoshu (DU 2) (2), Jizhong (DU 6) (2), Changqiang (DU 1) (2), Zhiyang (DU 9) (2), Shendao (DU 11) (2), Lingtai (DU 10) (1), Taodao (DU 13) (1)
RN	70	0.11	14	Qihai (RN 6) (14), Zhongwan (RN 12) (13), Lianquan (RN 23) (11), Guanyuan (RN 4) (11), Danzhong (RN 17) (6), Xiawan (RN 10) (5), Tiantu (RN 22) (2), Chengjiang (RN 24) (2), Juque (RN 14) (1), Shangwan (RN 13) (1), Shuifen (RN 9) (1), Yutang (RN 18) (1), Yinjiao (RN 7) (1), Zhongji (RN 3) (1)
GB	67	0.11	14	Yanglingquan (GB 34) (19), Fengchi (GB 20) (13), Zulinqi (GB 41) (6), Huantiao (GB 30) (5), Jianjing (GB 21) (5), Wangu (GB 12) (5), Fengshi (GB 31) (4), Xuanzhong (GB 39) (3), Jiaxi (GB 43) (1), Toulinqi (GB 15) (1), Xuanli (GB 6) (1), Qubin (GB 7) (1), Wushu (GB 27) (1), Qiuxu (GB 40) (1), Weidao (GB 28) (1)
SP	39	0.06	6	Sanyinjiao (SP 6) (20), Xuehai (SP 10) (8), Yinlingquan (SP 9) (7), Daheng (SP 15) (2), Gongsun (SP 4) (1), Taibai (SP 3) (1)
SJ	20	0.03	6	Waiguan (SJ 5) (10), Yifeng (SJ 17) (4), ZhongZhu (SJ 3) (3), Jianliao (SJ 14) (1), Yemen (SJ 2) (1), Tianjing (SJ 10) (1)
PC	17	0.03	4	Neiguan (PC 6) (14), Quze (PC 3) (1), Laogong (PC 8) (1), Tianchi (PC 2) (1)
KI	12	0.02	2	Taixi (KI 3) (11), Yongquan (KI 1) (1)
LR	11	0.02	2	Taichong (LR 3) (9), Zhangmen (LR 13) (2)
LU	8	0.01	5	Yuji (LU 10) (3), Chize (LU 5) (2), Zhongfu (LU 1) (1), Taiyuan (LU 9) (1), Yunmen (LU 2) (1)
SI	7	0.01	4	Tianzong (SI 11) (4), Houxi (SI 3) (1), Jianzhen (SI 9) (1), Tianrong (SI 17) (1)
HT	4	0.01	2	Jiquan (HT 1) (3), Shaofu (HT 8) (1)

**Table 2:** Frequency and percentage of acupoints in different body parts.

Distribution	Frequency	Percentage	Acupoint number	Acupoints (frequency)
Lower limbs	189	0.30	31	Zusanli (ST 36) (28), Sanyinjiao (SP 6) (20), Yanglingquan (GB 34) (19), Jiexi (ST 41) (11), Taixi (KI 3) (11), Futu (ST 32) (10), Taichong (LR 3) (9), Xuehai (SP 10) (8), Biguan (ST 31) (8), Liangqiu (ST 34) (8), Yinlingquan (SP 9) (7), Weizhong (BL 40) (6), Zulinqi (GB 41) (6), Huantiao (GB 30) (5), Kunlun (BL 60) (5), Fenglong (ST 40) (4), Fengshi (GB 31) (4), Xuanzhong (GB 39) (3), Tiaokou (ST 38) (2), Xiangu (ST 43) (2), Chengshan (BL 57) (2), Neiting (ST 44) (2), Chongyang (ST 42) (1), Shangjuxu (ST 37) (1), Dubi (ST 35) (1), Yongquan (KI 1) (1), Jiaxi (GB 43) (1), Gongsun (SP 4) (1), Taibai (SP 3) (1), Qiuxu (GB 40) (1), Heding (EX-LE2) (1)
Upper limbs	134	0.21	27	Hegu (LI 4) (28), Quchi (LI 11) (22), Neiguan (PC 6) (14), Shousanli (LI 10) (13), Jianyu (LI 15) (13), Waiguan (SJ 5) (10), Yangxi (LI 5) (4), Jiquan (HT 1) (3), Zhongzhu (SJ 3) (3), Yuji (LU 10) (3), Chize (LU 5) (2), Sanjian (LI 3) (2), Shouwuli (LI 13) (2), Binao (LI 14) (2), Pianli (LI 6) (1), Wenliu (LI 7) (1), Erjian (LI 2) (1), Shaofu (HT 8) (1), Quze (PC 3) (1), Laogong (PC 8) (1), Taiyuan (LU 9) (1), Houxi (SI 3) (1), Jianzhen (SI 9) (1), Jianliao (SJ 14) (1), Yemen (SJ 2) (1), Tianjing (SJ 10) (1), Baxie (EX-UE9) (1)
Back and lumbar	116	0.19	29	Jiaji (EX-B2) (15), Shenshu (BL 23) (14), Ganshu (BL 18) (13), Pishu (BL 20) (12), Weishu (BL 21) (6), Mingmen (DU 4) (6), Jianjing (GB 21) (5), Yaoyangguan (DU 3) (4), Tianzong (SI 11) (4), Feishu (BL 13) (4), Xinshu (BL 15) (4), Geshu (BL 17) (3), Shenshu (DU 12) (3), Zhongshu (DU 7) (2), Jinsuo (DU 8) (2), Zhiyang (DU 9) (2), Shendao (DU 11) (2), Jizhong (DU 6) (2), Changqiang (DU 1) (2), Yaoshu (DU 2) (2), Pohu (BL 42) (1), Shentang (BL 44) (1), Guanyuanshu (BL 26) (1), Ciliao (BL 32) (1), Zhibian (BL 54) (1), Geguan (BL 46) (1), Dingchuan (EX-B1) (1), Lingtai (DU 10) (1), Taodao (DU 13) (1)
Head, face, and neck	113	0.18	32	Fengchi (GB 20) (13), Baihui (DU 20) (12), Dazhui (DU 14) (12), Lianquan (RN 23) (11), Shuigou (DU 26) (6), Shenting (DU 24) (5), Jinjin (EX-HN12) (5), Yuye (EX-HN13) (5), Wangu (GB 12) (5), Fengfu (DU 16) (4), Tianshu (BL 10) (4), Yifeng (SJ 17) (4), Yamen (DU 15) (3), Sishencong (EX-HN1) (2), Tiantu (RN 22) (2), Chengjiang (RN 24) (2), Futu (LI 18) (1), Jiache (ST 6) (1), Touwei (ST 8) (2), Xiaguan (ST 7) (2), Quepen (ST 12) (1), Luoque (BL 8) (1), Tongtian (BL 7) (1), Chengguang (BL 6) (1), Wuchu (BL 5) (1), Xuanli (GB 6) (1), Qubin (GB 7) (1), Toulinqi (GB 15) (1), Tianrong (SI 17) (1), Yintang (EX-HN3) (1), Juquan (EX-HN10) (1), Shanglianquan (EX-HN 20) (1)
Chest and abdomen	74	0.12	22	Qihai (RN 6) (14), Zhongwan (RN 12) (13), Guanyuan (RN 4) (11), Danzhong (RN 17) (6), Xiawan (RN 10) (5), Tianshu (ST 25) (4), Qichong (ST 30) (3), Zhangmen (LR 13) (2), Daheng (SP 15) (2), Wailiing (ST 26) (1), Huaroumen (ST 24) (2), Zhongji (RN 3) (1), Shangwan (RN 13) (1), Shuifen (RN 9) (1), Juque (RN 14) (1), Yutang (RN 18) (1), Yinjiao (RN 7) (1), Wushu (GB 27) (1), Weidao (GB 28) (1), Zhongfu (LU 1) (1), Yunmen (LU 2) (1), Tianchi(PC 1) (1)

## Data Availability

The data during the current study are available from the corresponding author on reasonable request.

## Supplementary Materials

Supplementary Materials Supplementary Appendix 1: search strategy used in the PubMed database. Supplementary Appendix 2: acupoint combination by means of complex network analysis (weight ≥30). Supplementary Appendix 3: acupoint combination by means of association rule analysis for acupoints (>10 times).
