# Supplementary material for: Analysis on Acupoint Selection and Combination for Amyotrophic Lateral Sclerosis Treated with Acupuncture Based on Data Mining
Source: Evid Based Complement Alternat Med. 2022 Jun 8;2022:6541600. doi: 10.1155/2022/6541600 (PMC9200494; doi:10.1155/2022/6541600)
Supplement: Supplementary Materials — Supplementary Appendix 1: search strategy used in the PubMed database. Supplementary Appendix 2: acupoint combination by means of complex network analysis (weight ≥30). Supplementary Appendix 3: acupoint combination by means of association rule analysis for acupoints (>10 times). [file 6541600.f1.pdf]

Appendix 1: Search strategy used in PubMed database

#1 randomized controlled trial [pt]

#2 controlled clinical trial [pt]

#3 clinical trials [MeSH]

#4 randomized [tiab]

#5 trial [ti]

#6 randomly [tiab]

#7 placebo [tiab]

#8 #1 OR #2 OR #3 OR #4 OR #5 OR #6 OR #7

#9 humans [MeSH]

#10 #8 and #9

#11 amyotrophic lateral sclerosis [MeSH]

#12 motor neuron disease [MeSH]

#13 (ALS or MND): ti,ab

#14 #11 or #12 or #13

#15 acupuncture therapy (MeSH)

#16 (acupuncture or electroacupuncture or manual acupuncture or acupoint or meridian or scalp acupuncture or elongated acupuncture or abdominal acupuncture): ti,ab

#17 #15 or #16

#18 #10 and #14 and #17

Appendix 2: Acupoint combinations by means of complex network analysis (weight≥30).

Order	Acupoint Combinations		Weight
1	Hegu (LI 4)——	Zusanli (ST 36)	75
2	Quchi (LI 11)——	Zusanli (ST 36)	66
3	Quchi (LI 11)——	Hegu (LI 4)	65
4	Zusanli (ST 36)——	Sanyinjiao (SP 6)	54
5	Hegu (LI 4)——	Sanyinjiao (SP 6)	54
6	Quchi (LI 11)——	Sanyinjiao (SP 6)	49
7	Qihai (RN 6)——	Hegu (LI 4)	40
8	Hegu (LI 4)——	Yanglingquan (GB 34)	40
9	Zusanli (ST 36)——	Yanglingquan (GB 34)	40
10	Qihai (RN 6)——	Zusanli (ST 36)	40
11	Hegu (LI 4)——	Jianyu (LI 15)	38
12	Zusanli (ST 36)——	Jiexi (ST 41)	38
13	Zusanli (ST 36)——	Jianyu (LI 15)	38
14	Hegu (LI 4)——	Jiexi (ST 41)	38
15	Pishu (BL 20)——	Zusanli (ST 36)	37
16	Qihai (RN 6)——	Quchi (LI 11)	36
17	Shenshu (BL 23)——	Zusanli (ST 36)	36
18	Quchi (LI 11)——	Jiexi (ST 41)	35
19	Ganshu (BL 18)——	Zusanli (ST 36)	35
20	Quchi (LI 11)——	Jianyu (LI 15)	35
21	Hegu (LI 4)——	Zhongwan (RN 12)	34
22	Pishu (BL 20)——	Hegu (LI 4)	34
23	Zusanli (ST 36)——	Zhongwan (RN 12)	34
24	Neiguan (PC 6)——	Zusanli (ST 36)	34
25	Guanyuan (RN 4)——	Zusanli (ST 36)	33
26	Neiguan (PC 6)——	Hegu (LI 4)	33
27	Qihai (RN 6)——	Sanyinjiao (SP 6)	33
28	Shousanli (LI 10)——	Zusanli (ST 36)	33
29	Shenshu (BL 23)——	Hegu (LI 4)	33
30	Hegu (LI 4)——	Futu (ST 32)	33
31	Guanyuan (RN 4)——	Hegu (LI 4)	33
32	Zusanli (ST 36)——	Futu (ST 32)	32
33	Ganshu (BL 18)——	Hegu (LI 4)	32
34	Quchi (LI 11)——	Zhongwan (RN 12)	32
35	Shousanli (LI 10)——	Hegu (LI 4)	32
36	Guanyuan (RN 4)——	Quchi (LI 11)	31
37	Quchi (LI 11)——	Yanglingquan (GB 34)	31
38	Quchi (LI 11)——	Futu (ST 32)	31
39	Pishu (BL 20)——	Quchi (LI 11)	31
40	Yanglingquan (GB 34)——	Sanyinjiao (SP 6)	31
41	Waiguan (SJ 5)——	Hegu (LI 4)	30
42	Hegu (LI 4)——	Jiaji (EX-B2)	30
43	Sanyinjiao (SP 6)——	Zhongwan (RN 12)	30
44	Zusanli (ST 36)——	Jiaji (EX-B2)	30

Appendix 3: Acupoint combinations by means of association rule analysis from acupoints (> 10 times).

Acupoint	Combinations	Support	Confidence	Lift
Quchi (LI 11)——	Hegu (LI 4), Zusanli (ST 36)	0.52	1	1.5
Sanyinjiao (SP 6)——	Hegu (LI 4), Zusanli (ST 36)	0.43	0.9	1.35
Shousanli (LI 10)——	Hegu (LI 4), Zusanli (ST 36)	0.31	1	1.5
Jianyu (LI15), Shousanli (LI10)——	Hegu (LI 4)	0.31	1	1.5
Ganshu (BL 18)——	Shenshu (BL 23)	0.31	1	3.0
Qihai (RN 6)——	Sanyinjiao (SP 6)	0.31	0.93	1.95
Shenshu (BL 23)——	Zusanli (ST 36)	0.31	0.93	1.39
Shenshu (BL 23)——	Ganshu (BL 18)	0.31	0.93	3.0
Shousanli (LI10), Jianyu (LI15)——	Quchi (LI 11)	0.29	0.92	1.76
Jianyu (LI 15), Ganshu (BL 18)——	Zusanli (ST 36)	0.29	0.92	1.38
Jiexi (ST 41)——	Quchi (LI 11)	0.26	1	1.91
Jiexi (ST 41)——	Hegu (LI 4), Zusanli (ST 36)	0.26	1	1.5
Guanyuan (RN 4)——	Hegu (LI 4), Zusanli (ST 36)	0.26	1	1.5
Jiexi (ST41), Guanyuan (RN4)——	Zusanli (ST 36)	0.26	1	1.5
Pishu (BL 20)——	Zusanli (ST 36)	0.26	0.92	1.38
Taixi (KI 3)——	Sanyinjiao (SP 6)	0.26	1	2.1
Taixi (KI 3)——	Zusanli (ST 36)	0.26	1	1.5
Waiguan (SJ 5)——	Hegu (LI 4), Zusanli (ST 36)	0.24	1	1.5
Futu (ST 32)——	Quchi (LI 11)	0.24	1	1.91
Waiguan (SJ 5), Futu (ST 32)——	Zusanli (ST 36)	0.24	1	1.5
Futu (ST 32)——	Hegu (LI 4), Zusanli (ST 36)	0.24	1	1.5
Guanyuan (RN 4)——	Sanyinjiao (SP 6)	0.24	0.91	1.91
Guanyuan (RN 4)——	Quchi (LI 11)	0.24	0.91	1.74
Guanyuan (RN 4)——	Qihai (RN 6)	0.24	0.91	2.73
Waiguan (SJ 5)——	Sanyinjiao (SP 6)	0.21	0.90	1.89
Waiguan (SJ 5)——	Yanglingquan (GB 34)	0.21	0.90	1.99
Waiguan (SJ 5)——	Quchi (LI 11)	0.21	0.90	1.72
